# GR Dimerization and the Impact of GR Dimerization on GR Protein Stability and Half-Life

**DOI:** 10.3389/fimmu.2019.01693

**Published:** 2019-07-17

**Authors:** Ann Louw

**Affiliations:** Department of Biochemistry, Stellenbosch University, Stellenbosch, South Africa

**Keywords:** glucocorticoid receptor dimerization, acquired glucocorticoid resistance, Compound A, GR^dim^ mutant, GR^mon^ mutant, ubiquitin proteasomal system, biased ligands, half-life

## Abstract

Pharmacologically, glucocorticoids, which mediate their effects via the glucocorticoid receptor (GR), are a most effective therapy for inflammatory diseases despite the fact that chronic use causes side-effects and acquired GC resistance. The design of drugs with fewer side-effects and less potential for the development of resistance is therefore considered crucial for improved therapy. Dimerization of the GR is an integral step in glucocorticoid signaling and has been identified as a possible molecular site to target for drug development of anti-inflammatory drugs with an improved therapeutic index. Most of the current understanding regarding the role of GR dimerization in GC signaling derives for dimerization deficient mutants, although the role of ligands biased toward monomerization has also been described. Even though designing for loss of dimerization has mostly been applied for reduction of side-effect profile, designing for loss of dimerization may also be a fruitful strategy for the development of GC drugs with less potential to develop GC resistance. GC-induced resistance affects up to 30% of users and is due to a reduction in the GR functional pool. Several molecular mechanisms of GC-mediated reductions in GR pool have been described, one of which is the autologous down-regulation of GR density by the ubiquitin-proteasome-system (UPS). Loss of GR dimerization prevents autologous down-regulation of the receptor through modulation of interactions with components of the UPS and post-translational modifications (PTMs), such as phosphorylation, which prime the GR for degradation. Rational design of conformationally biased ligands that select for a monomeric GR conformation, which increases GC sensitivity through improving GR protein stability and increasing half-life, may be a productive avenue to explore. However, potential drawbacks to this approach should be considered as well as the advantages and disadvantages in chronic vs. acute treatment regimes.

## Introduction

Pharmacologically, glucocorticoids are a cost-effective effective therapy for inflammatory and autoimmune diseases and are widely prescribed (1–3). Despite the effectiveness of glucocorticoids in treating inflammation chronic use causes side-effects (4) and acquired glucocorticoid resistance (5, 6). The design of drugs with fewer side-effects and less potential for the development of resistance is therefore considered crucial for improved therapy (7).

**Figure 1:**
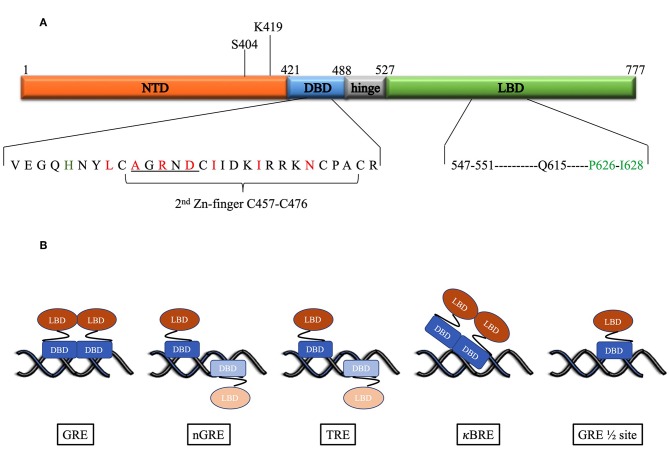
**(A)** Domain structure of the human GR. Above the figure is indicated the position of the post-translational modifications required for proteasomal degradation. Below the figure the DBD and LBD residues involved in the dimer interface are expanded. For the DBD, the underlined residues indicate the D-box, while red residues are those identified as important for the dimerization interface by Luisi et al. (8). In addition, in green is H472 in the lever arm that adopts one of two conformations: packed or flipped depending on whether binding to GREs or nGREs occur. For the LBD black residues are those involved in hydrogen bonds, while the green residues form hydrophobic interactions to stabilize the dimer interface as identified by Bledsoe et al. (9). **(B)** DNA-binding motifs determine orientation and GR monomer vs. dimer binding. Faded monomer indicates binding to low affinity site.

Glucocorticoids mediate their effects via the glucocorticoid receptor (GR) a ligand activated transcription factor. The GR has a domain structure that consists of an N-terminal domain (NTD), a DNA-binding domain (DBD) separated from the ligand binding domain (LBD) by a hinge region (Figure 1A) (10). The DBD contains two zinc fingers both of which are involved in DNA-binding, while the second zinc finger is also involved in dimerization. Binding of ligand to the LBD induces the cytoplasmic GR to dimerize and translocate to the nucleus where it can enhance transcription by binding cooperatively as a homodimer to glucocorticoid response elements (GREs), a consensus DNA sequence consisting of two hexameric half-sites separated by a 3-bp spacer. The monomeric GR can also repress transcription by binding directly to negative glucocorticoid response elements (nGREs) or GRE half-sites or by tethering to DNA-bound transcription factors such as NFκB or AP-1 (11–15).

The ability of the GR monomer to repress pro-inflammatory genes activated by NFκB or AP-1, while activating genes that result in the metabolic side-effects of glucocorticoids via the dimer binding to GREs suggested that separation of the transrepression and transactivation functions of the GR could give rise to safer drugs and resulted in the development of selective GR agonists (SEGRAs) or modulators (SEGRMs), collectively referred to as SEGRAMS (16–21). Despite the fact that the usefulness of this paradigm has been challenged as being outdated and oversimplifying the complexity of GR-signaling by negating the role of GR dimers in curbing inflammation and the role of GR monomers in eliciting side-effects (19, 22), it may still hold promise for drugs tailored to specific diseases phenotypes (18, 23, 24).

Although dimerization of the GR is an integral step in glucocorticoid signaling and fundamental to the concept of SEGRAMs it has only relatively recently been explicitly identified as a possible molecular site to target for drug development of anti-inflammatory drugs with an improved therapeutic index (23). In this review we thus discuss the identification of the GR dimerization interfaces, the use of GR dimerization mutants and conformationally biased ligands to further our understanding of the role of GR dimerization in GC signaling and the implications of loss of GR dimerization for reduction of side-effects, while highlighting the recent finding that loss of dimerization may also be a fruitful strategy for the development of drugs with less potential to develop glucocorticoid resistance.

## GR Dimerization

Although the ability of GR to form dimers in solution has been debated (8, 25–31) several studies have shown that the GR, liganded or unliganded, can dimerize in solution (32–36) and that dimerization may already be present in the cytoplasm (35, 37–39).

### X-Ray Crystallography of GR Domains Identifies Amino Acids Involved in Dimerization

Two interfaces in the GR have been identified that mediate receptor dimerization, the DBD and the LBD dimerization interfaces. Although no crystal structure of the full-length GR has been reported to date, separate crystal structures of the DBD and LBD have been reported, which identified specific amino acids involved in the dimerization interfaces and for the orientation of binding to DNA.

The first crystal structure of the rat GR DBD (amino acid residues 440–525) complexed to a canonical GR-binding element (GRE) identified a dimerization interface (Figure 1A) in the second zinc finger of the GR consisting of 7 amino acids (rat residues L475, A477, R479, D481, I483, I487, N491, which corresponds to the human residues L456, A458, R460, D481, I483, I487, N491) with three of the inter-subunit contacts in a region referred to as the D-box (C476–C482) (8). The two molecules of the DBD bind cooperatively to one face of the DNA (Figure 1B) when the two hexameric sites are separated by a 3-base pair spacer in a head-to-head fashion so that their dimerization loops (D-box) are aligned and contacting each other (8, 25). Furthermore, crystal structures of the DBD bound to different GREs were virtually super-imposable except for the lever arm, a loop region in the DBD between the DNA recognition helix (first zinc finger) and the dimerization loop, where different GREs dictate discrete alternate conformations (40). In addition, human residue H472 in the lever arm adopts one of two conformations: packed in the first monomer, which binds to the initial conserved half-site, and flipped in the second monomer, which binds to the second variable site in the GRE.

In contrast to the head-to-head binding of the DBD to GREs, crystal structures indicate that at a nGRE (Figure 1B), in the *TSLP* gene, which is like the canonical IR-GBS sequence: CTCC(n)_0−2_GGAGA (41), GR binds as two monomers orientated tail-to-tail in an everted repeat orientation on opposite sides of the DNA (42). This prevents DNA-mediated dimerization as the D-loops are directed away from each other and results in binding that is characterized by strong negative cooperativity, where binding of the first GR monomer to the high affinity site hampers binding of the second monomer to the low affinity site. The two-site binding event (Table 1) characterized by two non-identical, monomeric binding events has a lower binding affinity (363 nM and 63 μM) than positive cooperative binding to a GRE site (73 nM) (42). This suggests that the nGRE sequence not only preferentially binds GR monomers but that it contributes to a repressive conformation, which may involve a distinct lever arm conformation where H472 (rat residue) is flipped in both monomers (42). Crystal structures of GR DBD bound to AP-1 response elements (TREs: TGA(G/C)TC) (46) (Figure 1B) suggest a similar binding orientation and comparable binding affinities (Table 1). In contrast, crystal structures of GR DBD bound to NF-κB response (κBRE) elements (45) (Figure 1B) indicate that binding is head-to-head as for binding to the GREs but resembles those of the nGRE in that it presents with a two site-binding curve which, like for the nGRE (44), is abolished by the S425G human mutant. Although only one monomer binds to the conserved AATTY sequence (Y represents a pyrimidine base), it binds as a “D-loop” engaged dimer with high and low binding affinities in the same range as binding of the DBD to nGREs (Table 1). Collectively, the negative cooperativity of DNA binding as well as results with GR dimerization deficient mutants suggest that monomeric GR is likely sufficient at repressive GR binding elements (nGRE, TRE, and κBRE) *in vivo*. Occupancy of GR monomers at GRE half-sites has also been confirmed *in vivo* (14).

**Table 1:** DNA-binding affinity (${\text{K}}_{\text{d}}^{\text{a}}$) of domains and full-length wild-type and GR^dim^ dimerization deficient mutant (Hill-slope added in brackets).

		**GR^**wt**^**	**DBD mutant: ^b^GR^**dim**^**
DBD	GRE	•73 nM (42) •1.6 – 5.7 nM (1.8 – 2.1) (43) •80–890 nM (40) •73 nM (44) •5.7 nM (25) •7.14 – 25.7 nM (37)	•370 nM (42) •16 – 28 nM (1.3 – 1.4) (43)
	nGRE	•360 nM and 63 μM (42) •363 nM and 63.2 μM (44)	•1.1 μM (42)
	κBRE	•215 – 239 nM and 17 – >50 μM (45)	
	TRE	•12 – 402 nM and 1 – 12 μM (46)	
Full-length	GRE	•50 nM (36) •0.5 nM (25) •1.2 – 2.56 nM (37) •34 nM (46) •35 nM (45) •32 – 490 nM (47) •140 nM (2.5) (30)	•300 nM (36)
	κBRE	•51 nM (45)	
	TRE	•42 nM (46)	
	GRE $\frac{1}{2}$ sites	•1,210 nM (36) •185 nM (1.08) (14)	•1,260 nM (36)

*^a^ (K_app_), determined using the Langmuir binding model, is given as only some investigators (30, 47) determined K_tot_, the total affinity for assembling two GR monomers at the palindromic GRE*.

b *GR^dim^ = human GR^A458T^, mouse GR^A465T^, and rat GR^A477T^*.

Comparison of initial structural studies of the free GR DBD solved by NMR (48–51) with that of the crystal structure of DNA bound GR DBD (8) suggested that the largest difference occurred in the D-box and led to the assumption that DNA binding was required for dimerization. However, comparison of a recent crystal structure of the free human GR DBD (residues 418–517) (52) with that of previously determined crystal structures of the GR DBD bound to a GRE or a nGRE reveal a very similar core structure with a similar D-loop conformation and indicates that the largest difference is located in the lever arm. Molecular dynamic simulations of the lever arm suggest that it is most mobile in the free state sampling the most diverse number conformations, while in the nGRE-bound state an intermediate number of conformations are present, which is further reduced in the GRE-bound state. Thus, binding to DNA constrains the number of conformations that the lever arm can sample, which is further reduced upon dimerization, however, the D-loop is accessible in solution for dimerization via the DBD.

The crystal structure of the GR LBD lagged behind because of solubility problems, however introduction of a single mutation (human residue F602S) significantly improved solubility without affecting function and allowed for crystallization of the LBD (human residues 521–777) in the presence of ligand dexamethasone (DEX) and TIF2, a coactivator peptide (9). This led to the identification of a dimerization interface (Figure 1A) stabilized by hydrophobic interactions, specifically reciprocal interactions between P625 and I628 in the H5–H6 loop, and hydrogen bonds, from particularly residues between 547 and 551 (extended strand between helices 1 and 3) and Q615 (last residue in helix 5) from each LBD, that allows formation of four hydrogen bonds (9). Subsequent GR LBD crystal structures (53–58) in the presence of agonist or antagonist, focused mainly on the ligand-binding pocket rather than on the dimerization interface and generally conform to the crystal structure of the Bledsoe group (9), besides identifying differences in the ligand-binding pocket and helix 12. Recently Bianchetti et al. (59) evaluated the physiological relevance of the GR LBD dimerization interface by analyzing 20 published GR LBD crystal structures using estimates of dimer stability (surface area in Å^2^ buried upon dimerization and estimated free energy variation (Δ^i^G) upon formation of the interface) coupled to evolutionary sequence conservation analysis of the interface. One GRα LBD homodimer structure, the apH9 dimer, consistently stood out as being more stable, by having the largest contact surface area (850Å^2^) and the lowest binding free energy variation upon formation of the interface (Δ^i^G: −42.9 kcal/mol), and as having highly (82%) conserved residues at the interface (27 of the 33 residues that contributed to binding were conserved), however, this structure was formed by only one of the crystal structures investigated (PDB ID:4P6W) (53). In contrast, the other dimerization structures observed in GR LBD crystals were less stable and not significantly conserved, with the bat-like structure for the GR LBD, suggested by Bledsoe et al. (9), which was observed in 6 PDB entries (28%) (9, 53–55, 57, 58), being amongst the least stable (surface area buried is 288Å^2^ and Δ^i^G: −20 kcal/ mol) and conserved (7/16 = 44%), while the most frequent H1 structure, observed in 9 entries (43%) (9, 53–58), had a slightly higher stability (332Å^2^ and Δ^i^G −30 kcal/ mol) and lower number of conserved residues (2/5) (59). In summary, this suggests that the GR LBD dimers are generally weaker and less conserved than the nuclear receptor LBD dimer through H9-H10-H11 (also called the butter-fly like structure with 1494Å^2^ and Δ^i^G: −77.5 kcal/ mol and 73% of conserved residues at the interface), which is found in the ER LBD, a sentiment supported by Billas and Moras (60). Despite the fact that the bat-like dimer structure was found to be physiologically the least stable by Bianchetti et al., of the residues suggested to be important for stabilization of the dimer interface, three residues involved in the hGR hydrophobic interface core (Y545 in H1-loop-H3, P625 in S1-turn-S2 and I628 in S2) and one (Gln 630 in H5) identified as part of the hydrogen-bond network, were previously identified by Bledsoe et al. (9). Interestingly, the surface area buried originally reported for the bat-like structure (1623Å^2^) by Bledsoe et al. (9) is much higher than that reported by Bianchetti et al. (59) (288Å^2^) for this structure.

### GR Dimerization Mutants Confirm Role of GR Dimerization Interfaces

Genetic strategies have also been used to verify the GR interfaces involved in dimerization and the relevance of specific amino acids identified from crystal structures. Although, these GR dimerization deficient mutants have been studied extensively for their role in the regulation of gene expression (12, 61–63), here mainly effects on dimerization will be discussed.

#### Mutants That Target the DBD

Most of the GR dimerization mutation studies focused of the DBD dimerization interface (64), specifically the three amino acids in the D-loop (Figure 1A), with the GR^dim^ mutant (human GR^A458T^, mouse GR^A465T^, and rat GR^A477T^) the most widely characterized and extensively studied (64–66). A backbone hydrogen bond is formed between the carbonyl of A777 and the amide of I483 on the associated dimer partner (8) and mutation of the Ala to Thr has been shown disrupt this interaction (43, 65, 66).

##### Effects on dimerization

There has been much controversy surrounding the dimerization potential of the GR^dim^ mutant with several publications suggesting that dimerization equal to that of GR^wt^ occurs. Most of the studies showing similar dimerization as the GR^wt^ were semiquantitative: co-immunoprecipitation (62) and Numbers & Brightness (N&B) assay (31).

However, quantitative studies at the single-cell level, using fluorescence correlation spectroscopy (FCS) combined with a microwell system, have shown that GR^dim^ has a dissociation constant (K_d_) of dimerization (Table 2) in the presence of DEX that is only slightly lower than that of the GR^wt^ in the absence of ligand [370 nM for GR^dim(+DEX)^ vs. 410 nM GR^wt(−DEX)^ *in vitro* (36) and 6.11 μM for GR^dim(+DEX)^ vs. 7.4 μM for GR^wt(−DEX)^ *in vivo* (35)], but significantly higher than that of GR^wt^ in the presence of DEX [370 nM for GR^dim(+DEX)^ vs. 140 nM GR^wt(+DEX)^ *in vitro* (36) and 6.11 μM for GR^dim(+DEX)^ vs. 3 μM for GR^wt(+DEX)^ *in vivo* (35)]. This indicates that the dimerization potential of the mutant GR^dim^ is substantially lower than that of the GR^wt^ in the presence of DEX and closer to the dimerization potential of GR^wt^ in the absence of ligand. Although it is evident that the GR^dim^ can form dimers, it is also clear that the monomer-dimer equilibrium of the mutant is shifted in the direction of monomers and it is clearly deficient in dimerization potential when compared to GR^wt^.

**Table 2:** Dimerization dissociation constants (K_d_) of domains and full-length wild-type and select mutant GRs (^a^Method used and DEX concentration in brackets).

	**GR^**wt**^**	**DBD mutant:**	**LBD mutant:**
		**^b^GR^**dim**^**	**^c^GR^**I628A**^**
DBD	•13 – 21 nM (EMSA) (37)		
LBD	**Liganded:**•1.5 μM (AU; 10 μM) (9)		**Liganded:**•15 μM (AU; 10 μM) (9)
Full-length	**Unliganded:**•410 nM (FCS) (36) •3.9 nM (EMSA) (37) •100 μM (AU) (30) •416 nM (FCS) (35) •7.4 μM (FCS^*^) (35)	**Unliganded:** •390 nM (FCS) (36)	
	**Liganded:**•140 nM (FCS; 500 nM) (36) •139 nM (FCS; 100 nM) (35) •3 μM (FCS^*^; 100 nM) (35) •107 nM (FCS; 500 nM) (67)	**Liganded:** •370 nM (FCS; 500 nM) (36) •379 nM (FCS; 100 nM) (35) •6.11 μM (FCS^*^; 100 nM) (35)	

a *Methods to determine dimerization*: •EMSA, electrophoretic mobility shift assay •AU, analytic ultracentrifugation •FCS, fluorescence correlation spectroscopy (only method also done in intact live cells and indicated as FCS^*^).

b *GR^dim^ = human GR^A458T^, mouse GR^A465T^, and rat GR^A477T^*.

c *human GR^I628A^, mouse GR^I634A^, and rat GR^I646A^*.

The dimerization equilibrium may also be influenced by receptor concentration. At low concentrations of GR (335 fmol/mg protein or 26200 GR/cell) the extent of DEX-induced dimerization of GR^dim^ (37%) is much less than that of the GR^wt^ (100%), but similar to that of uninduced GR^wt^ (43%), while at about a 4-fold higher receptor concentration (1,420 fmol/mg protein or 111,000 GR/cell), the extent of DEX-induced dimerization of GR^dim^ (90%) approaches that of the induced GR^wt^ (100%) and uninduced GR^wt^ (102%) (38).

##### Effects on DNA binding

Binding to diverse GR binding motifs could also support dimer vs. monomer GR conformations especially if the Hill-slope^1^ is reported as a measure of cooperativity (Table 1). Positive cooperative DNA-binding requires binding of a GR dimer, where binding of the first monomer facilitates binding of the second monomer, and exhibits an increased binding affinity with a Hill-slope larger than 1. Although it was initially reported that the GR^dim^ could not bind to DNA (65, 66) it is now clear that maximal DNA-binding of the GR^dim^ mutant, both as DBD and as full-length receptor, to a GRE is not affected (43). However, the mutant binds with a lower affinity (Table 1) (36, 42, 43). Furthermore, the A477T mutant dissociates faster that the wild type receptor (5–12x faster *in vitro* for DBD with a dissociation half-life (t12) of 23–55 s for GR^wt^ vs. 4.7–4.8 s for the GR^dim^ (43) and 10x faster *in vivo* for the full-length receptor with a residence time for GR^wt^ that is 1.45 s vs. 0.15 s for GR^dim^ (68) due to a reduction, but not abrogation, in positive cooperative DNA binding (Hill-slope for GR^wt^ 1.8–2.1 and for GR^dim^ 1.3–1.4) (43). Interestingly, in addition to GR^dim^, other salt bridge mutations (rat GR^R479D^ or GR^D481R^) disrupting the DBD dimer interface also result in lower binding to a single GRE but higher binding to paired GREs and thus enhanced transcriptional synergy at reiterated GREs (69–71).

Comparison of binding affinities of the GR^wt^ to that of GR^dim^ to other GR DNA-binding motifs (Table 1) is also informative in terms of probing a more monomeric binding configuration for GR^dim^. Thus, although GR^dim^ substantially decreases the overall affinity of the DBD for a GRE, for a nGRE, it binds with a similar affinity as the GR^wt^ binding to a nGRE (42). Furthermore, the full-length receptor GR^dim^ mutant binds to a GRE half-site with an equivalent affinity as that of the GR^wt^ (36). Additionally, ChIP-exo in liver and in primary bone marrow–derived macrophages (15) or human U2OS osteosarcoma cell lines (14, 72) indicates that GR^wt^, but not GR^dim^, binds to GRE sequences as a dimer, while both receptors bind to tethered and half-site motifs as monomers.

#### Mutants That Target the LBD

There is a paucity of GR dimerization mutation studies focusing on the LBD dimerization interface, most probably as this dimerization interface was characterized (9) almost 10-years later than that of the DBD interface (8). Although the dimerization affinity of the liganded human GR LBD (1.5 μM) is already low in comparison to that of the DBD or the full-length receptor (Table 2), it was reduced 10-fold by the LBD mutant, hGR^I628A^, which displays a phenotype very similar to that of the GR^dim^ mutant (9). However, in contrast, using the N&B assay it was shown that the mouse GR^I634A^ mutant displayed reduced dimerization relative to GR^wt^ and GR^dim^ at equivalent DEX concentrations, suggesting that the LBD plays a potentially larger role than the DBD in GR dimerization (31). Furthermore, a combination mutant involving both the DBD and LBD domains (mGR^A465T/I634A^ called GR^mon^) has recently been described and comparison of the dimerization potential with that of liganded GR^wt^ and single mutants using N&B assays indicate that the order of DEX dimerization efficiency is GR^wt^ = GR^dim^ > GR^I634A^ > GR^mon^, however, at higher DEX concentration (1 μM) significant dimerization of the GR^mon^ is still seen (31).

### Small Molecules Displaying Loss of GR Dimerization (Conformationally Biased Ligands)

Despite the fact that one would assume that the search for SEGRAMs would have yielded several small molecule ligands that perturb the GR monomer-dimer equilibrium as the concept is underpinned by the idea that targeting for loss of GR dimerization would reduce the side-effect profile (23), it appears that the guiding principle in this search has rather been to assay for a preference to induce transrepression rather than transactivation and that very few SEGRAMs have been evaluated for their effects on GR dimerization (18, 73–77). Two conformationally biased ligands that perturb the GR monomer-dimer equilibrium have, however, been identified: CpdA (Compound A: 2-(4acetoxyphenyl)-2- chloro-*N*-methylethylammonium chloride), an analog of a naturally occurring compound found in the Namibian shrub *Salsola tuberculatiformis* Botsch (78), and 21-hydroxy-6,19-epoxyprogesterone (21OH-6,19OP), a progesterone derivative (79, 80).

**Figure 2:**
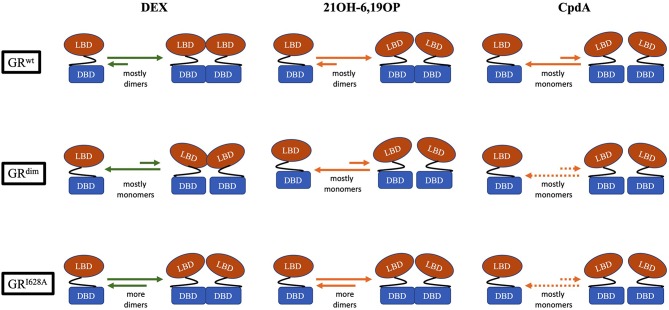
Schematic representation of the monomer-dimer equilibrium for GR^wt^, the DBD-dimerization deficient mutant, GR^dim^, and the LBD-dimerization deficient mutant, GR^I628A^, bound to either, DEX, 21OH-6,19OP, or CpdA. In the equilibrium, green arrows represents quantitative data, while orange arrows represents semiquantitative or qualitative data (see Table 2). Dotted orange arrows represents hypothesized equilibria not yet determined.

CpdA not only prevents dimerization of the full-length GR^wt^ receptor *in vitro* and *in vivo* (Figure 2), but abrogates basal (uninduced) GR dimerization (31, 38, 81, 82). In contrast, 21OH-6,19OP does not prevent dimerization of the full-length GR or the LBD dimerization mutant, GR^I634A^ (Figure 2), but does prevent dimerization of the DBD GR^dim^ mutant, suggesting that it prevents dimerization via the LBD (31), which is supported by molecular dynamics simulations that suggests this ligand triggers a conformational change in the H1–H3 loop dimerization interface that differs substantially from that induced by DEX (83).

Despite the fact that it is clear that the GR monomer-dimer equilibrium may be modulated by changes in receptor and ligand concentrations (31, 38), by dimerization deficient mutants (31, 66) and by conformationally biased ligands (80, 81), there is still a controversy regarding the relative contributions of the DBD (60, 84) and LBD (31) to dimerization of the full-length receptor and whether other regions, such as the hinge region (39) and the N-terminal-domain (37), play a substantial role in dimerization. In addition, it seems unlikely that a single point mutation in either the DBD or the LBD would fully abrogate the ability of the GR to dimerize. Quantitative analysis in live cells (29) comparing the dimerization affinity of different GR dimerization mutants, such as done for GR^dim^ (35, 36), could, however, help to resolve the relative contributions of point mutations to the dimerization potential of the GR. Dimerization assays in intact live cells clearly deliver dimerization affinity constants that differ significantly from those obtained in cell lysates as seen in the study of Tiwari et al. (35), where for example, the K_d_ of dimerization of the liganded GR^wt^ is significantly lower *in vitro* (139 nM) than *in vivo* (3 μM) (Table 2). The most parsimonious explanation for this phenomenon entails that an increase in free GR monomer concentration or a decrease in free dimer concentration occurs *in vivo* after ligand-binding, which would be sufficient to favor a higher K_d_^2^. In support of this, it has recently been suggested that in mouse livers the GR binds predominantly as a monomer under physiological conditions but that after addition of exogenous glucocorticoid there is a ligand-dependent redistribution of GR from monomer to dimer at GR binding sites (15), thus effectively decreasing free dimer and increasing free monomer concentrations in the nucleus. Furthermore, the implications of higher order GR tetramers bound to DNA, that are produced from GR dimers preformed in the nucleoplasm, recently described (29, 85), in terms of the GR monomer-dimer equilibrium still remains to be elucidated as do the individual amino acids involved in this interaction.

## Impact of GR Dimerization on the Therapeutic Index of Glucocorticoids

Despite their wide-spread use the therapeutic index (TI^3^) of glucocorticoids remains low (86), especially in the chronic long-term (>6 months), high-dose (>2.5–10 mg/day) scenario (87, 88), with side-effects (4, 89, 90) and loss of glucocorticoid sensitivity or glucocorticoid resistance (5, 91), respectively, affecting the numerator and denominator of the TI.

The discussion in this section will focus on *in vivo* studies of loss of GR dimerization achieved using either the GR^dim^ mutation or CpdA. 21OH-6,19OP, which affects dimerization of only the LBD and as such does not affect dimerization of the full-length GR^wt^ receptor (31), was originally described as a specific passive antiglucocorticoid (92, 93) but displays dissociated activity *in vivo* (94), However, as very few *in vivo* studies (79, 80) have been conducted this molecule will not be discussed further.

### Glucocorticoid-Induced Side-Effects

Evaluation of the impact of GR dimerization on glucocorticoid signaling has focused mainly on the modulation of the side-effect profile elicited by glucocorticoids (23, 95).

Generally, loss of GR dimerization, whether through the use of the GR^dim^ mutant and/or the GR^dim/dim^ mouse model (66), or the monomeric favoring ligand, CpdA, has resulted in effective inflammatory control with a reduction in side-effects (96–98). For example, in a recent systemic review comparing the efficacy and safety of SGRMs to that of glucocorticoids in arthritis it was found that CpdA generally displays an improved TI with a similar efficacy but a better safety profile than glucocorticoids (17).

To illustrate, the effect of loss of GR dimerization on two side-effects of systemic use of glucocorticoids for severe asthma in the UK with an increased hazard ratio (HR), namely diabetes (HR:1.20) and osteoporosis (HR: 1.64) (99), will be discussed. Diabetogenic effects, which include increased blood glucose levels, gluconeogenesis, glycogen storage, insulin secretion and/or liver metabolic enzyme transcription are mediated by GR transactivation and requires GR dimerization, were not observed with GR^dim^ (63, 100, 101) or with CpdA (82, 97, 102–104). While, osteoporosis, mediated by both transrepression (osteocalcin transcription) and transactivation (osteoblast differentiation) and thus requiring both GR monomers and dimers (95), was not induced by CpdA, either *in vitro* or *in vivo* (105–109), while the GR^dim^ mice still developed osteoporosis concomitant with a potent suppression of osteoblast differentiation both *in vitro* and *in vivo* (110–112).

Interestingly, loss of GR dimerization through use of GR^dim^ mice also appears to limit gastrointestinal side-effects of DEX such as enhanced glucose transport in the small intestine (63) and an increase in gastroparesis (delayed stomach emptying) and gastric acid secretion (113). However, some side-effects of glucocorticoids still occur in GR^dim^ mice (95, 114). For example, DEX induced a similar degree of atrophy in the tibilialis anterior and gastrocnemius muscles of GR^wt^ and GR^dim^ mice (115). Investigation involving a key regulator of muscle atrophy, the E3-ubiquitin ligase, MuRF1, suggests that GR-binding is stabilized by the binding of an adjacent FOXO1 on a composite DNA-binding element in the proximal promotor of the gene, as GR^dim^ alone, in contrast to GR^wt^, did not induce the MuRF1 promoter but did result in a modest induction in the presence of FOXO1, which itself is upregulated by DEX via GR^wt^ (116), but not GR^dim^ (115). CpdA has not been evaluated in this model and it would be interesting to establish if, like for osteoporosis, loss of dimerization through CpdA administration has a more favorable outcome than seen with GR^dim^. Tantalizingly, in the *mdx* mouse model of Duchenne muscular dystrophy CpdA, unlike prednisolone, did not reduce gastrocnemius muscle mass (117).

However, as an important caveat it should be noted that loss of GR dimerization through the GR^dim^ mutation can impair the effect of glucocorticoid treatment in some inflammatory conditions and as discussed may still display some DEX-induced side-effects (95, 114). For example, in skin, inhibition of the swelling response during the challenge phase, upon re-exposure to the hapten, 2,4-dinitrofluorobenzene, by exogenous intra-peritoneal or oral DEX administration in contact dermatitis, a T cell–dependent delayed-type hypersensitivity reaction, is not observed in GR^dim^ mice (118), yet in phorbol ester-induced inflammation, a classic model of acute irritant inflammation and epidermal hyperplasia, topical DEX-treatment was as effective in GR^dim^ mice (96). For CpdA, results in acute irritant inflammation of the skin are conflicting and may depend on the topical dose used. At low doses [μg range (119, 120)] CpdA not only inhibited irritant-induced skin inflammation and hyperplasia but also did not induce skin atrophy, an important side-effect of topical glucocorticoid treatment. However, at higher doses (mg range) CpdA increased, rather than decreased, epidermal thickness (121).

In two models of arthritis in mice, antigen-induced arthritis (AIA), a mouse model of human rheumatoid arthritis, and glucose-6-phosphate isomerase-induced arthritis, a severe form of polyarthritis, GR^dim^ mice were, respectively, fully or partly resistant to intravenous Micromethason (liposomal encapsulated DEX) treatment (122). In contrast, CpdA administered intraperitoneally showed similar or slightly reduced efficacy compared to DEX in attenuating collagen-induced arthritis (82, 123, 124) and repressed the inflammatory response as effectively as glucocorticoids in *ex-vivo* models using fibroblast-like synoviocytes (FLS) from rheumatoid arthritis or osteoarthritis patients (108, 123, 125, 126), while displaying less side-effects, such as hyperinsulinemia (82), bone-loss (108, 124) and homologous down-regulation of the GR (123), than glucocorticoids.

Both GR^dim^ (127) and CpdA (104, 128) was as effective as DEX treatment in experimental autoimmune encephalomyelitis, a mouse model of multiple sclerosis, while CpdA, unlike DEX, did not elicit hyperinsulinemia or hypothalamic-pituitary-adrenal axis suppression (104). However, in allergic airway inflammation (AAI), a mouse model of allergic asthma, GR^dim^ mice, unlike GR^wt^ mice, did not respond to intraperitoneal injection of DEX (129), while CpdA was as effective as DEX in this model (130).

In acute systemic inflammatory settings GR^dim^ mice are highly vulnerable and resistant to glucocorticoid treatment. For example, in two mouse models of sepsis, cecal ligation and puncture and lipopolysaccharide (LPS)-induced septic shock, GR^dim^ mice are highly susceptible to sepsis and their bone marrow-derived macrophages are resistant to DEX treatment *in vitro* (131). Interestingly, even low dose LPS treatment resulted in GR^dim^ mice displaying exaggerated sickness behavior compared to GR^wt^ mice (132). Furthermore, in TNF-induced acute lethal inflammation GR^dim^ mice displayed increased TNF sensitivity and resistance to DEX treatment (133, 134). Acute graft- vs.-host disease, a severe complication of hematopoietic stem cell transplantation, is another severe inflammatory disease characterized by a cytokine storm in which GR^dim^ mice presented with exacerbated clinical symptoms and increased mortality relative to GR^wt^ (135). To our knowledge CpdA has not been evaluated in these acute inflammatory models although it has been suggested that it would be as ineffective as the GR^dim^ mice as for full resolution of the inflammatory response dimerization of the GR is required [22, 23].

In addition, concerns regarding specifically the use of CpdA as a therapeutic agent have been raised (102, 124, 128, 130) as it degrades to an aziridine in solution (78) thus mediating cytotoxic effects independent of the GR that may severely narrow its therapeutic window.

### Glucocorticoid-Induced Resistance

Glucocorticoid resistance is characterized by impaired sensitivity to glucocorticoid treatment and may be inherited (136) or acquired, which is more common and may result from disease progression or chronic high-dose glucocorticoid treatment (5, 91). One of the main drivers of acquired glucocorticoid resistance is homologous down-regulation of the GR (5, 137, 138).

Mechanism-based pharmacodynamic models use the term drug tolerance to describe the decrease in expected pharmacological response after repeated or continuous drug exposure (139) and modeling of the pharmacogenomic responses of glucocorticoid-induced leucine zipper (GILZ) (140) and tyrosine aminotransferase (TAT) (141) mRNA induction by both acute and chronic glucocorticoid regimes in diverse rat tissues indicate that drug tolerance is primarily controlled by the cytosolic free receptor density, which is substantially down-regulated.

Receptor density is modulated by *de novo* receptor synthesis and receptor degradation, which may be described by a simple “push” vs. “pull” mechanism (5), where the “push” mechanism includes transcription initiation and mRNA stability, while the “pull” mechanism involves degradation of the receptor.

Already 30 years ago, it was established that ligand-mediated down-regulation of the GR occurs at the level of both transcription initiation and GR protein degradation, but not at the level of mRNA stability (142). Further elucidation of the process has established that inhibition of transcription is mediated through binding of the liganded-GR to a nGRE in exon 6 of the GR gene and assembly of a repressive complex, consisting of the GR, the coregulator NCoR1, and histone deacetylase 3 (HDAC3), at the transcriptional start site through DNA-looping (143), while ligand-dependent GR protein degradation has been localized to the ubiquitin-proteasome system (UPS) through the use of the proteasome inhibitors (144). Proteasomal degradation requires ligand-induced phosphorylation of the human GR at S404 (Figure 1A) by glycogen synthase kinase 3β (GSK3β) (145), which is required for ubiquitination of the human GR at the upstream K419 (mouse GR K426) in a PEST sequence (144, 146). Ubiquitin is attached to the GR in a three step pathway involving ubiquitin activating (E1), conjugating (E2), and ligase (E3) enzymes to produce a polyubiquitylated receptor for targeting to the 26S proteasome (147). Several E2-conjugating enzymes, such as ubiquitin-conjugating enzyme 7 (UbcH7) (148), susceptibility gene 101 (TSG101) (149), and Ubc9 (150–152) and E3-ligases, such as E6-AP (encoded by the *Ube3a* gene) (153, 154), carboxy-terminus of heat shock protein 70-interacting protein (CHIP)(155–157), murine (Mdm2), or human (Hdm2) double minute (158–160), UBR1 (161), and F-box/WD repeat-containing protein 7 (FBXW7α) (162), have been shown to interact with the GR. Recently, however, micoRNAs (miRNAs), upregulated by glucocorticoids (163, 164), have been implicated in the ligand-induced reduction of the GR mRNA pool (5, 10), suggesting that the initial study indicating that receptor density is not regulated by the stability of mRNA levels has to be re-examined.

The relative contributions of GR mRNA and protein down-regulation may be dependent on the dose of glucocorticoid and/or the duration of treatment. For example, in podocytes GR protein, but not RNA, is down-regulated during both short (1 h) high (100 μM) dose and long-term (5 days) low (1 μM) dose DEX regimes (165), while in HeLa S3 cells, 24 h, 2 weeks or a 2-year low (1 μM) dose DEX regime suggests that at 24 h, GR protein is more profoundly down-regulated than mRNA, while at 2 weeks both protein and mRNA is down-regulated, while by 2-years no detectable protein or RNA was observed (166). Furthermore, in FLS derived from patients with rheumatoid arthritis a short (7 h) vs. long (30 h) protocol of low (1 μM) dose DEX indicates substantially more GR protein down-regulation at the longer time point (123).

Although little to no work has been done on the implications of GR dimerization for GR resistance, some tantalizing results with GR ligands have been noted. For example, RU486 (mifepristone), a GR antagonist shown to cause significantly less dimerization than DEX (167), was unable to down-regulate nascent GR RNA (143) and was less effective than DEX at down-regulating GR protein levels (168), while ZK216348, a SEGRA (169) for which no data on GR dimerization is available, did not down-regulate GR protein levels (102). CpdA, which abrogates GR dimerization (31, 81, 82, 170), does not result in GR down-regulation at either protein (102, 123, 171–175) or RNA (123, 172) level.

Recently, our laboratory investigated the hypothesis that GR dimerization may be required for homologous down-regulation of the GR by employing conditions that either promote or reduce GR dimerization (176). Promotion of GR dimerization through the use of dimerization promoting ligands, such as DEX and cortisol, induced significant down-regulation of GR^wt^, both transiently transfected and endogenous in HepG2 cells, while reduction of dimerization, through the use of either CpdA or GR^dim^, severely restricted GR turn-over. Receptor down-regulation was primarily mediated by increasing the rate of receptor protein turnover by the proteasome as (1) promotion of GR dimerization significantly increased the rate of turnover and decreased receptor half-life relative to the unliganded receptor and (2) inhibition of the proteasome by MG132, but not protein synthesis by cycloheximide, abolished GR turn-over. Interestingly, the GR^wt^ half-life with CpdA was very similar to that of the half-life of the unliganded receptor, a finding previously reported (171). Mechanistically, degradation of the GR by the proteasome requires hyperphosphorylation of the GR at S404 by GSK3β (145), which enables binding of the E3 ligase FBXW7α (162). Loss of GR dimerization restricted hyperphosphorylation at S404 and interaction with FBXW7α. Furthermore, inhibition of DEX-mediated S404 hyperphosphorylation through the use of the pharmacological GSK3β inhibitor, BIO, restored GR levels. In summary, GR dimerization is required for ligand-induced post-translational processing and downregulation of the receptor via the UPS system. Subsequently, the requirement of GR dimerization for autologous down-regulation of the GR was confirmed in a study in arthritic mice indicating that DEX does not down-regulate the GR in GR^dim^ mice, in contrast to GR^wt^ mice (164).

Although, loss of GR dimerization has been generated by using either dimerization deficient mutants such as GR^dim^, or monomerization biased ligands such as CpdA, and it has been suggested that the behavior of DEX-induced GR^dim^ equates to that of CpdA-induced GR^wt^ (81), results show that the two scenarios do not always produce exactly the same results. At a molecular level, for example, although both GR^dim^ and CpdA prevent homologous down-regulation of the GR the two conditions differ in terms of the extent of the repression of the post-translational modifications (PTMs) required for the process, with CpdA reducing S404 phosphorylation, while no discernible, not even basal, phosphorylation is observed with GR^dim^ (176). Nuclear translocation of the GR is another area of potential difference as some studies show that CpdA does not allow for nuclear translocation of the GR^dim^ (176), while others suggest that both GR^dim^ and CpdA can cause nuclear translocation albeit with diminished maximal import (81, 170). Furthermore, in disease models, although glucocorticoid-induced metabolic side-effects may be attenuated under both conditions, GR^dim^ can still induce osteoporosis, while CpdA does not, which has been ascribed to the ability of GR^dim^, but not CpdA, to suppress interleukin-11 via interaction with AP-1 (108, 111, 177). Additionally, in terms of efficacy in disease models loss of dimerization through CpdA administration often had a more favorable outcome than seen with GR^dim^ mice, in for example, arthritis (82, 108, 122–126) and allergic asthma (128–130) models. Although it may be tempting to ascribe these differences to the extent of GR dimerization elicited, with total abrogation of dimerization by CpdA (31, 81) and no (31, 62), to partial (38), to almost full (35, 36) loss of dimerization via GR^dim^, this would probably be an oversimplification. More likely is that CpdA, in contrast to GR^dim^ that impacts only the DBD (65), also elicits a differential conformation of the LBD upon binding (97), which could impact on GR PTMs (97, 171, 176) and interaction with cofactors (178, 179). Despite the fact that both CpdA and GR^dim^ modulate GR dimerization there are few comparative studies directly comparing implications for molecular aspects of GR signaling or the impact on the therapeutic index in mouse models of disease.

## Conclusion

Monomeric GR, like the dimer, binds to DNA and is transcriptionally functional (101), thus these two receptor species may represent distinct drug targets to tailor for improved glucocorticoid treatments. Rational design of conformationally biased ligands that select for a monomeric GR conformation, may be a productive avenue to explore in the pursuit of drugs that lessen the side-effect profile and increase glucocorticoid sensitivity through improving GR protein stability and increasing half-life, yet the optimal conformational and gene expression signatures to either drive the monomer-dimer equilibrium toward a particular state or evaluate its implications remain elusive, as does the question of whether this would be feasible or even desirable in the clinic.

For rational structure-based drug optimization strategies the field needs to look at both methods to accurately measure and quantify GR dimerization bias and an updated theoretical framework or model to evaluate the implications of GR dimerization.

Biased signaling is well-developed in the field of GPCR signaling (180) and offers quantification approaches (181) that yield useful empirical parameters, such as the transduction coefficient (τ/K_A_) that incorporates ligand efficacy and potency as well as receptor density, to compare extent of bias relative to a reference ligand, usually the endogenous ligand (182). However, in the GR field there have been only isolated reports that harnessed classical analytical pharmacology approaches to generate quantitative information about the pharmacodynamic properties of GR ligands (183, 184). In addition, although mechanistic pharmacokinetic and pharmacodynamic models for the GR (140, 185, 186) and mathematical models to increase drug specificity (187–189) are being developed their uptake by most investigators has been slow. This is unfortunate as they provide a much-needed new perspective and are an essential component for understanding the quantitative behavior of biased GR ligands and to provide tractable design strategies such as functional selectivity fingerprints for drug development.

**Figure 3:**
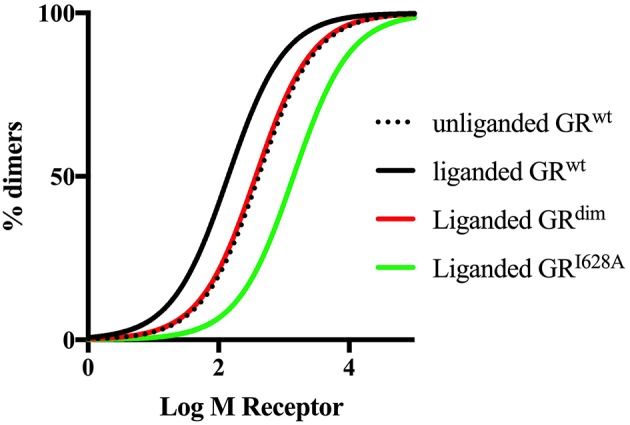
Simulated dimerization curves for unliganded and liganded GR^wt^ and liganded GR^dim^ and GR^I628A^. Simulations were done using GraphPad Prism version 7. K_d_ values from Oasa et al. (36) were used, except for liganded GR^I628A^, where a 10-fold increase in the K_d_ of the unliganded GR^wt^ was used as per Bledsoe et al. (9). The figure clearly shows that ligand-binding to the GR^wt^ results in a left shift of the dimerization curve, while mutations in either the DBD or the LBD dimerization interfaces result a right shift of the curve relative to GR^wt^, with a more pronounced shift in the case of the mutation to the LBD dimerization interface.

The importance of quantitative, rather than semiquantitative analysis is illustrated by the recent commotion around the usefulness of the GR^dim^ model to investigate effects of loss of dimerization. The initial study by Presman et al. (31) using the N&B assay that demonstrated dimerization by the GR^dim^ was semiquantitative yet several reviews since then have given this evidence underserved prominence. Mass action dictates that increasing GR levels would force the steady state to dimerization even in the case of a GR species poorly able to elicit dimerization, such as the GR^dim^. Thus, a valid evaluation and comparison of the dimerization potential of the GR^dim^ requires a quantitative approach that measures dimerization affinity such as done by the group of Kinjo (35, 67). Furthermore, it has recently been pointed out that the N&B assay may suffer from drawbacks, which could be avoided by using the two-detector number and brightness analysis (TD-N&B) (190), whereby it was shown that the GR^dim^ is poorly dimerized in the nucleus, with a concentration ratio between monomers and dimers of 1:0.66 as compared to GR^wt^ that has a concentration ratio between monomers and dimers of 1:19.1. Finally, simulated dimerization curves using the K_d_ values obtained from the literature (Figure 3) clearly shows that the GR^dim^ is indeed poor at eliciting dimerization in comparison to GR^wt^.

Despite optimism regarding the potential of biased ligands such as SEGRMs to improve on the therapeutic potential of glucocorticoids, to date none have entered the market (191). For biased ligands promoting GR monomers there are indeed legitimate concerns raised that for full resolution of inflammation transactivation by GR-dimers of genes such as mitogen-activated protein kinase phosphatase-1 (MKP-1), GC-induced leucine zipper (GILZ), and IL10 are required (22). Notwithstanding these concerns a strong argument has been made for the tailoring of ligands that favor GR monomer formation for chronic long-term use (23), a scenario where the additional ability of these ligands to prevent resistance would be most relevant.

## Author Contributions

The author confirms being the sole contributor of this work and has approved it for publication.

### Conflict of Interest Statement

The author declares that the research was conducted in the absence of any commercial or financial relationships that could be construed as a potential conflict of interest.

## Footnotes

^1^If the Hill slope is = 1, binding is additive, if >1, binding displays positive cooperativity, while if >1, binding displays negative cooperativity.

^2^(K_d_ = $\frac{\left[GR\;monomer\right]x\left[GR\;monomer\right]\;}{\left[GR\;dimer\right]}$).

^3^$TI=\;\frac{TD50\;\left(dose\;of\;drug\;that\;causes\;severe\;side\;effects\;in\;50\%\;of\;subjects\right)}{EC50\;\left(dose\;of\;drug\;that\;has\;desired\;pharmacologivcal\;effect\;in\;50\%\;of\;subjects\right)}$
